# Whole-Genome Sequences of Antibiotic-Resistant Trueperella pyogenes Isolates from Surgical Site Infections in Dairy Cows in Switzerland

**DOI:** 10.1128/mra.00865-22

**Published:** 2022-11-15

**Authors:** Emma Marchionatti, Vincent Perreten

**Affiliations:** a Clinic for Ruminants, Vetsuisse Faculty, University of Bern, Bern, Switzerland; b Division of Molecular Bacterial Epidemiology and Infectious Diseases, Institute of Veterinary Bacteriology, Vetsuisse Faculty, University of Bern, Bern, Switzerland; University of Rochester School of Medicine and Dentistry

## Abstract

The complete genome sequence of four Trueperella pyogenes isolated from cattle surgical site infections in Switzerland was determined using hybrid assembly of Oxford Nanopore and Illumina reads. Genes conferring resistance to tetracyclines [*tet*(W)], sulfonamides (*sul1*), chloramphenicol (*cmx*), streptomycin/spectinomycin (*aadA1*), and quaternary ammonium compounds (*qacE*Δ*1*) were identified on different chromosomal elements.

## ANNOUNCEMENT

Trueperella pyogenes is a Gram-positive, non-spore-forming, nonmotile, noncapsulated, facultative anaerobic rod belonging to the biota of the skin and mucous membranes of the upper respiratory, urogenital, and gastrointestinal tracts of animals. *T. pyogenes* is also an opportunistic pathogen causing suppurative infections in animals and rarely in humans (1). The infections are commonly treated with antibiotics, posing the risk of selection of resistant bacteria through the acquisition of mobile genetic elements containing antimicrobial resistance genes (2).

The complete genome sequence was determined for *T. pyogenes* strains EMSSI21, EMSSI48, EMSSI54, and 22KM0800 obtained from surgical site infections of dairy cows. The strains were isolated on Trypticase soy agar containing 5% defibrinated sheep blood (TSA-S) (Becton Dickinson) after 5% CO_2_ incubation at 37°C for 24 h, identified as *T. pyogenes* by matrix-assisted laser desorption ionization–time of flight (MALDI-TOF) mass spectrometry (Bruker), and cryopreserved in our collection. MICs were determined following CLSI recommendations (3). All strains showed decreased susceptibility to tetracycline (MIC of ≥16 μg/mL) and, except for EMSSI21, to streptomycin (MIC of 32 μg/mL) and sulfamethoxazole (MIC of ≥512 μg/mL). EMSSI48 also exhibited decreased susceptibility to chloramphenicol (MIC of 8 μg/mL).

Genomic DNA was extracted from a lawn of colonies grown overnight on TSA-S at 37°C under 5% CO_2_ using a MasterPure complete DNA and RNA purification kit (Lucigen). A complete circular genome was obtained by hybrid assembly of long and short reads from Oxford Nanopore Technologies (ONT) and Illumina. Illumina reads were obtained from a NEBNext Ultra II directional DNA library with TruSeq adapters on an Illumina NovaSeq 6000 system (2 × 150-bp paired-end reads) (NGS Platform, University of Bern, Switzerland; Eurofins Genomics, Germany). Illumina sequences were quality filtered and paired using Trimmomatic v.0.36 (4). ONT DNA libraries were obtained from unsheared DNA using the ligation kit SQK-LSK109, loaded onto a FLO-MIN106D flow cell R9.4.1, and sequenced using a MinION Mk1B device (ONT). Base calling and demultiplexing were performed using Guppy v.4.4.1 (ONT) (5), and ends were trimmed and sizes filtered using Cutadapt v.2.5 (6). Illumina reads were used without filtering after confirmation of high-quality and adapter-free sequences by FastQC v.0.11.7 analysis (7). ONT results were visualized using the controlling software MINKNOW-GUI v.19.05.0. The genome was *de novo* assembled, circularized, and rotated to start at DnaA using Unicycler v.0.4.4 run in bold mode with paired-end Illumina reads and ONT reads larger than 10 kb (8). Default parameters were used for all software unless otherwise specified. Genome annotation was done by the NCBI Prokaryotic Genome Annotation Pipeline (9). Sequencing statistics and genomic characteristics are presented in Table 1.

**TABLE 1:** Sequencing statistics and genomic and antimicrobial resistance gene characteristics of *T. pyogenes* strains EMSSI21, EMSSI48, EMSSI54, and 22KM0800 from surgical site infections in dairy cows^*a*^

Parameter	Result for strain (accession no.):			
	EMSSI21 (CP081508)	EMSSI48 (CP096280)	EMSSI54 (CP096279)	22KM0800 (CP097247)
Illumina statistics (7)				
Read length (bp)	151	139–151	137–151	151
No. of reads	13,396,826	1,769,981	1,995,732	6,530,649
Oxford Nanopore statistics (11)				
*N*_50_ (bp)	25,144	12,969	11,189	11,390
Mean read length (bp)	12,166	8,528	6,274	7,719
No. of reads	9,069	107,621	83,946	16,914
Genomic characteristics				
Total length (bp)	2,253,850	2,223,178	2,224,507	2,301,561
GC content (%)	59.5	59.8	59.7	59.5
No. of:				
Predicted genes	2,051	1,198	2,007	2,075
CDSs	1,993	1,940	1,948	2,017
Pseudogenes	16	17	17	18
rRNAs	3	3	3	3
tRNAs	46	46	47	46
ncRNAs	3	3	3	3
Antibiotic resistance features				
Gene positions				
*tet*(W)	1571634–1569715	1525397–1523478	1554018–1552099	1584244–1582325
*aadA1*	NP	2121408–2122244	1813183–1812347	1852907–1852071
*sul1*	NP	2122749–2123588	1811842–1811003	1851566–1850727
*qacE*	NP	2122408–2122755	1812183–1811836	1851907–1851560
*cmx*	NP	2124482–2125657	NP	NP

Element(s)	ATE-1 [*tet*(W)]	ATE-1 [*tet*(W)]	ATE-1 [*tet*(W)]	ATE-1 [*tet*(W)]
	NP	IS*6 intI1 aadA1 qacE*Δ*1 sul1* GNAT *N*-acetyltransferase *cmx* IS*481* IS*6*	IS*6* GNAT *N*-acetyltransferase *sul1 qacE*Δ*1 aadA1* transcriptional regulator IS*6*	IS*6* GNAT *N*-acetyltransferase *sul1 qacE*Δ*1 aadA1* transcriptional regulator IS*6*

a Abbreviations: CDSs, coding DNA sequences; ncRNAs, noncoding RNAs; NP, not present in the genome.

All strains carried the tetracycline ribosomal protection protein gene *tet*(W) in association with the genetic element ATE-1 (10). All strains but EMSSI21 carried the streptomycin/spectinomycin adenylyl transferase gene *aadA1*, the sulfonamide-resistant dihydropteroate synthase gene *sul1*, and the quaternary ammonium compound efflux gene *qacE*Δ*1*. Two IS*6* copies flanked the composite element containing the resistance genes. EMSSI48 also carried the chloramphenicol efflux MFS transporter gene *cmx* (Table 1).

These genomes permitted the detection of resistance mechanisms associated with antibiotics commonly used in cattle and will serve as basis for further whole-genome sequencing (WGS)-based studies and nosocomial outbreak investigations.

### Data availability.

The complete genome sequences of *T. pyogenes* strains EMSSI21, EMSSI48, EMSSI54, and 22KM0800 have been deposited in GenBank under accession no. CP081508, CP096280, CP096279, and CP097247, respectively. The associated BioProject and BioSample accession no. are PRJNA755484, PRJNA826253, and PRJNA834611 and SAMN20826602, SAMN27553866, and SAMN27553867, SAMN28052907, respectively. The raw reads were deposited in the SRA database under accession no. SRR15507453, SRR18740466, SRR18740464, and SRR19049134 (Illumina) and SRR15507454, SRR18740467, SRR18740465, and SRR19049135 (ONT).
